# MneA and TerC exhibit distinct roles in manganese detoxification and virulence in *Vibrio parahaemolyticus*

**DOI:** 10.1128/aem.01754-25

**Published:** 2026-05-20

**Authors:** Chengkun Zheng, Yu Zhang, Xiaoya Zhu, Furong Ma, Jiawen Liu, Zhengzhong Xu, Xinan Jiao, Xiang Chen

**Affiliations:** 1 Jiangsu Key Laboratory of Zoonosis/Jiangsu Co-Innovation Center for Prevention and Control of Important Animal Infectious Diseases and Zoonoses, Yangzhou University 38043, Yangzhou, China; 2 Key Laboratory of Prevention and Control of Biological Hazard Factors (Animal Origin) for Agri-food Safety and Quality, the Ministry of Agriculture and Rural Affairs, Yangzhou University 38043, Yangzhou, China; 3 Joint International Research Laboratory of Agriculture and Agri-Product Safety, Ministry of Education, Yangzhou University 38043, Yangzhou, China; Indiana University Bloomington, Bloomington, Indiana, USA

**Keywords:** *Vibrio parahaemolyticus*, manganese detoxification, virulence, MneA, TerC

## Abstract

*Vibrio parahaemolyticus* is a significant zoonotic pathogen that severely threatens global public health and aquaculture. Manganese (Mn) acts as a cofactor for numerous enzymes and is crucial for bacterial oxidative stress resistance and virulence. However, excessive Mn is toxic, prompting bacteria to develop diverse Mn detoxification mechanisms. Herein, two genes (*mneA* and *terC*) located downstream of the Mn-sensing *yybP-ykoY* riboswitch were identified in *V. parahaemolyticus*. The expression of *mneA* and *terC* is specifically induced by Mn. The *mneA* deletion mutant (Δ*mneA*) exhibited impaired growth in standard (pH 7.3), alkaline (pH 8.4), or weakly acidic (pH 6.8) medium supplemented with Mn. By contrast, the *terC* deletion mutant (Δ*terC*) showed growth inhibition under high Mn concentrations in weakly acidic medium. Furthermore, the *mneA* and *terC* double mutant (Δ*mneA* Δ*terC*) was more sensitive to Mn stress than Δ*mneA*. Bacterial survival assays revealed that both MneA and TerC protect *V. parahaemolyticus* against Mn-induced bacteriostasis. Inductively coupled plasma-mass spectrometry showed that the deletion of *mneA* or *terC* significantly increases intracellular Mn content. A competitive infection assay using a zebrafish model revealed that *mneA* contributes to virulence in *V. parahaemolyticus*, while *terC* has no such effect. RNA sequencing analysis revealed that *V. parahaemolyticus* modulates the expression of multiple genes in response to Mn bacteriostatic activity. Collectively, these findings indicate that MneA and TerC exhibit distinct roles in Mn detoxification and virulence in *V. parahaemolyticus*. This work expands the understanding of Mn detoxification mechanisms in bacteria and highlights the role of Mn in the host–pathogen interface.

**IMPORTANCE:**

Manganese (Mn) is important for life, but is toxic in excess. Consequently, bacteria have evolved elaborate mechanisms to maintain intracellular Mn homeostasis. In this study, we identified and characterized two Mn exporters, that is, MneA and TerC, in *Vibrio parahaemolyticus*, a significant zoonotic pathogen. We showed that MneA and TerC protect *V. parahaemolyticus* against Mn-induced bacteriostasis via Mn efflux. Strikingly, unlike the findings in *Escherichia coli* that a TerC homolog Alx tunes intracellular Mn concentration at alkaline pH, *V. parahaemolyticus* TerC facilitates the bacterium’s growth under high Mn concentrations in weakly acidic medium. Moreover, MneA is crucial for the virulence of *V. parahaemolyticus*, whereas TerC plays no apparent role. Collectively, while both MneA and TerC act as Mn exporters in *V. parahaemolyticus*, they exhibit distinct roles. This study also highlights the importance of exploring conserved proteins in different bacterial species.

## INTRODUCTION

*Vibrio parahaemolyticus* is a gram-negative halophilic bacterium that is extensively distributed across temperate and tropical marine and coastal regions worldwide (1). First isolated in Japan in 1950, it is now recognized as a significant foodborne pathogen and a leading cause of seafood-related gastroenteritis, typically resulting from the consumption of raw or undercooked contaminated seafood (2–4). While infections are often self-limiting, they may progress to severe and potentially fatal sepsis, particularly in immunocompromised individuals or those with underlying conditions such as liver disease or diabetes (5). Additionally, *V. parahaemolyticus* can also cause wound infections (6). From 1990 to 2019, *V. parahaemolyticus* accounted for more than 40 global outbreaks, second only to *Vibrio cholerae* in terms of outbreak frequency (7). Beyond its impact on human health, *V. parahaemolyticus* can also cause aquatic animal diseases, such as acute hepatopancreatic necrosis syndrome in shrimp, thereby leading to substantial economic losses to the global aquaculture industry (8).

Transition metals, such as iron (Fe), manganese (Mn), zinc (Zn), and copper (Cu), are broadly needed for life. Except for Zn, these metals have unfilled *d*-orbitals, endowing them with redox activity (9). They are frequently integrated into metal proteins, such as metalloenzymes, storage proteins, and transcription factors, thereby regulating protein structure or associated catalytic reactions (10). It is estimated that approximately one-third of the entire proteome is metal-binding proteins (11). In the context of infection, transition metals play a central role in the battle between the host and pathogens. Vertebrate hosts have evolved strategies to inhibit bacterial proliferation by imposing metal restrictions or metal toxicity through nutritional immunity (10). For instance, neutrophils mobilize Zn in response to *Streptococcus pyogenes* infection, potentially using it as an antimicrobial agent (12). Correspondingly, bacteria have evolved elaborate mechanisms to adapt to fluctuations in the levels of host metals, including metal-sensing transcription factors, metal importers and exporters, metal-detoxifying enzymes, and scavenger metalloproteins (13). Nevertheless, the mechanisms often vary among bacterial species or strains and are influenced by specific environmental conditions. For example, environmental and virulent *Francisella* species employ distinct Zn acquisition mechanisms (14), while group I and II strains of *Acidovorax citrulli* have different Cu tolerance mechanisms (15).

Mn is an essential nutrient involved in carbohydrate and nucleic acid metabolism, signal transduction, translation, and other physiological processes as a cofactor of various enzymes (16, 17). However, excessive Mn may disrupt the homeostasis of other metals, such as Zn, in cells (18). Consequently, some regulatory factors and enzymes may malfunction because of metal mismatching (19). In order to avoid Mn toxicity, bacteria have evolved various efflux systems to eliminate excessive Mn. Bacteria mainly regulate the expression of genes related to Mn homeostasis through two mechanisms: one involves Mn-binding transcription factors MntR/Mur and the other involves the Mn-binding riboswitch *yybP-ykoY* (20, 21). There are four main types of Mn efflux systems: the cation diffusion facilitator (CDF) (22, 23), MntP (24), UPF0016 (25), and P-type ATPase families (26). For example, in *V. cholerae*, the UPF0016 family MneA acts as a Mn exporter (25). Additionally, there is growing evidence that TerC family proteins participate in Mn detoxification (27–29). Notably, MeeF and MeeY, both members of the TerC family, are implicated in the metalation of exoenzymes during export (30). Mn efflux systems have been shown to be closely related to oxidative stress resistance or virulence in certain bacteria. For example, MntE, a member of the CDF family, is involved in Mn efflux in *Streptococcus pneumoniae*, and the deletion of *mntE* significantly attenuates virulence in mouse models (22). However, the mechanism for Mn detoxification in *V. parahaemolyticus* and its effect on the pathogenicity of this bacterium remain unclear.

This study sought to identify the Mn detoxification systems in *V. parahaemolyticus* and evaluate their role in bacterial virulence. We showed that while both MneA and TerC act as Mn exporters in *V. parahaemolyticus*, they exhibit distinct roles. MneA functions across varying pH conditions, whereas TerC operates in weakly acidic medium (pH 6.8). Moreover, MneA plays a crucial role in virulence.

## RESULTS

### Identification of potential Mn exporters MneA and TerC in *V. parahaemolyticus*

**Fig 1:**
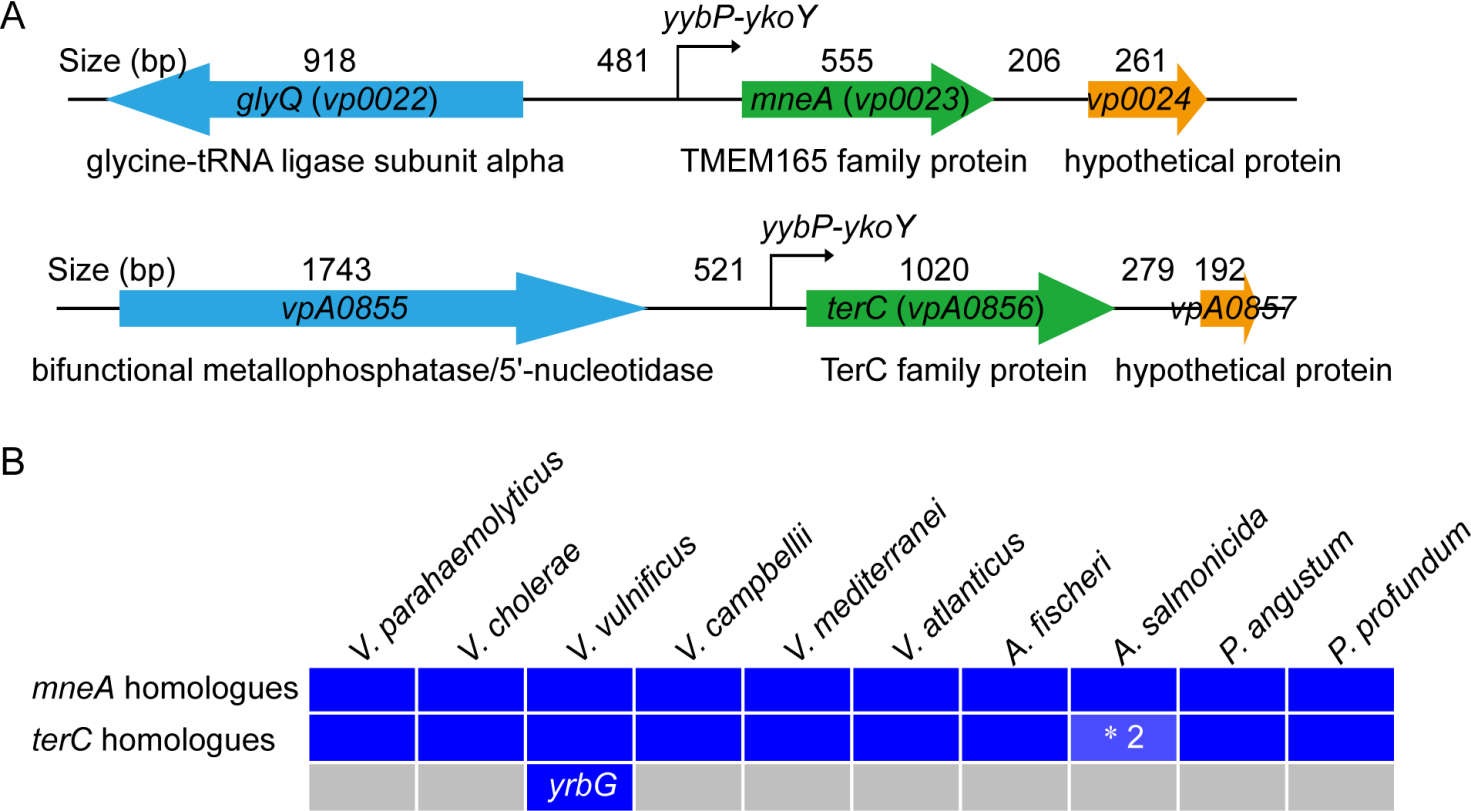
Bioinformatic analysis of the *mneA* and *terC* homologs. (**A**) The genetic structures of *mneA* and *terC* in *V. parahaemolyticus*. Arrows indicate the direction of transcription, numbers above the genes and intergenic regions indicate the sizes (bp). (**B**) The prevalence of the *mneA* and *terC* homologs in *Vibrionaceae*. The analysis was based on the following genomes: *V. parahaemolyticus* RIMD2210633, NC_004603.1; *V. cholerae* N16961, NC_002505.1; *V. vulnificus* CMCP6, NC_004459.3; *V. campbellii* ATCC BAA-1116, NC_009783.1; *V. mediterranei* AK1, NZ_ABCH00000000.1; *V. atlanticus* LGP32, NC_011753.2; *A. fischeri* ES114, CP000020.2; *A. salmonicida* LFI1238, NC_011312.1; *P. angustum* S14, GCA_000153265.1; and *P. profundum* SS9, NC_006370.1. The genome of *A. salmonicida* LFI1238 contains two copies of *terC* homologs.

Analysis with RegPrecise 3.2 (31) revealed that in *V. parahaemolyticus* RIMD2210633, the genes *VP0023* (designated as *mneA*) and *VPA0856* (designated as *terC*) are potentially regulated by the upstream *yybP-ykoY* riboswitch. *VP0023* encodes a TMEM165 family protein, whereas *VPA0856* encodes a TerC family protein (Fig. 1A). BLASTP analysis showed that the amino acid sequence of *V. parahaemolyticus* MneA shares 89.67% and 59.77% identity with MneA from *V. cholerae* (25) and *Pseudomonas* sp. NBB (32), respectively. In contrast, *V. parahaemolyticus* TerC exhibits only 41.90%, 38.40%, 30.10%, and 25.45% amino acid sequence identity with Alx of *Escherichia coli* (27), TerC of *Riemerella anatipestifer* (28), and YceF and YkoY of *Bacillus subtilis* (29), respectively. Additionally, the gene *VP1383* encodes a TerC family protein, which shows 23.76%, 25.45%, and 25.85% amino acid sequence identity with Alx of *E. coli* (27), and YceF and YkoY of *B. subtilis* (29), respectively. In contrast, no significant similarity was found between VP1383 and TerC from *V. parahaemolyticus* and *R. anatipestifer* (28). Importantly, no *yybP-ykoY* riboswitch was detected upstream of *VP1383*. Therefore, *VP1383* was not analyzed in the current study. The *mneA* and *terC* homologs are present in other species of *Vibrionaceae*, including *V. cholerae, Vibrio vulnificus*, *Vibrio campbellii*, *Vibrio mediterranei*, *Vibrio atlanticus*, *Aliivibrio fischeri*, *Aeromonas salmonicida*, *Photobacterium angustum*, and *Photobacterium profundum* (Fig. 1B). Notably, in *V. vulnificus*, the genes under the control of *yybP-ykoY* include *yrbG*, which encodes an inner membrane protein, and *A. salmonicida* possesses two copies of *terC* homologs (Fig. 1B). Multiple sequence alignment revealed that both the MneA and TerC homologs are highly conserved across *Vibrionaceae* (Fig. S1).

### The expression of *mneA* and *terC* is specifically induced by Mn

**Fig 2:**
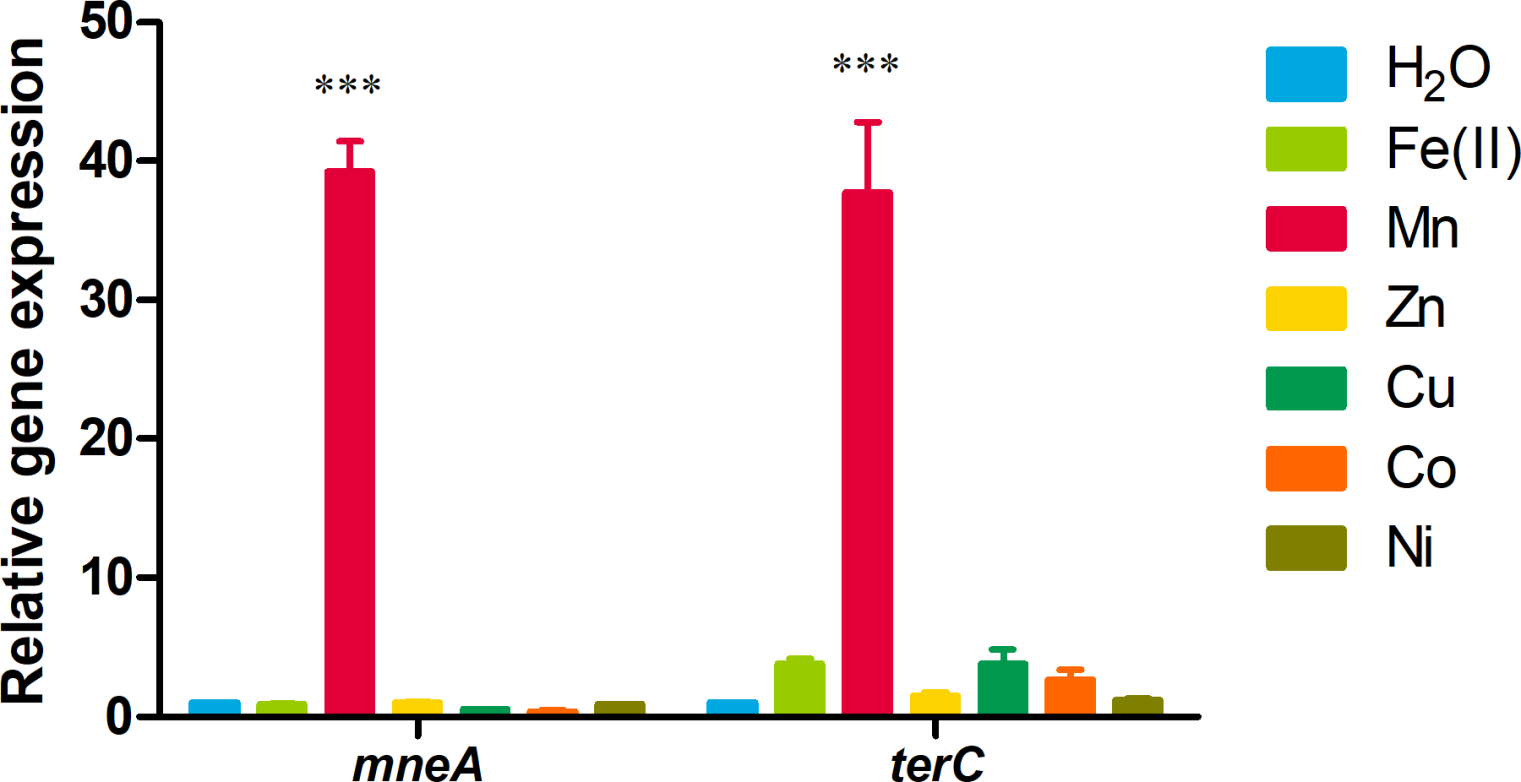
*V. parahaemolyticus* upregulates *mneA* and *terC* expression in response to Mn stress. The RIMD2210633 strain in the early exponential phase was treated with H_2_O (control), 1 mM FeSO_4_, 1 mM MnSO_4_, 0.5 mM ZnSO_4_, 1 mM CuSO_4_, 0.5 mM CoSO_44_, or 1 mM NiSO_44_ for 15 min. Total RNA was isolated, and quantitative real-time PCR was performed to assess *mneA* and *terC* expression relative to the control sample. The results represent the means and standard deviations from three independent experiments. The data were analyzed using one-way analysis of variance along with Bonferroni’s post-test. ***, *P*  <  0.001.

To explore the association of *mneA* and *terC* with metal homeostasis, we performed quantitative real-time PCR (qRT-PCR) to analyze the expression of these two genes under diverse metal stress conditions. As shown in Fig. 2, the expression levels of *mneA* and *terC* increased by approximately 39- and 38-fold, respectively, on treatment with 1 mM Mn. In contrast, compared with the control group, the expression levels of the two genes did not change significantly on treatment with other metals. These results indicate that *mneA* and *terC* may be involved in Mn homeostasis in *V. parahaemolyticus*.

### MneA promotes *V. parahaemolyticus* growth under elevated Mn concentrations in standard tryptic soy broth (TSB, pH 7.3)

In order to assess the role of *mneA* and *terC* in *V. parahaemolyticus*, we constructed mutants (Δ*mneA*, Δ*terC*, and Δ*mneA* Δ*terC*) and corresponding complemented strains (CΔ*mneA*, CΔ*terC*, and C(Δ*mneA* Δ*terC*)) based on *V. parahaemolyticus* RIMD2210633 (33) and evaluated their growth curves under metal stress conditions.

**Fig 3:**
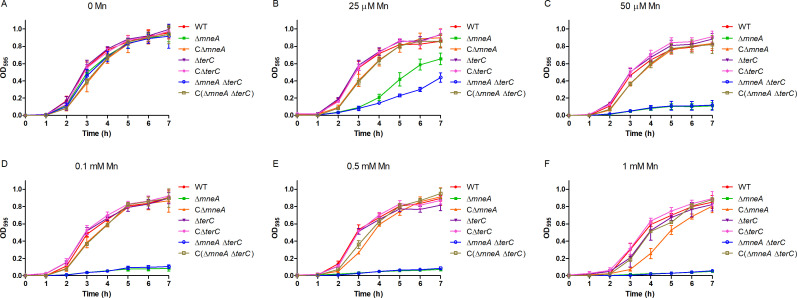
The effect of added Mn on the growth of the *V. parahaemolyticus* strains in standard TSB. The *V. parahaemolyticus* strains were grown in standard TSB supplemented with 0 μM (**A**), 25 μM (**B**), 50 μM (**C**), 0.1 mM (**D**), 0.5 mM (**E**), or 1 mM (**F**) Mn. For the complemented strains, chloramphenicol (25 μg/mL) and IPTG (1 mM) were added to the medium to maintain the plasmid and induce gene expression. The WT and mutant strains were grown without chloramphenicol and IPTG. The results represent the means and standard deviations from three independent experiments performed in duplicate.

We evaluated the growth of the wild-type (WT), mutant, and complemented strains in standard TSB supplemented with Mn or other metals. In the absence of metal supplementation, the growth curves of the WT, mutant, and complemented strains were almost identical (Fig. 3A). However, when 25 μM Mn was added, the growth of Δ*mneA* and Δ*mneA* Δ*terC* was obviously inhibited, with Δ*mneA* Δ*terC* being more strongly inhibited. The growth trend of CΔ*mneA* and C(Δ*mneA* Δ*terC*) was similar to that of the WT strain (Fig. 3B). As the Mn concentrations increased further, the Δ*mneA* and Δ*mneA* Δ*terC* mutants were almost completely inhibited (Fig. 3C through F). When the Mn concentration reached 1 mM, CΔ*mneA* was slightly inhibited (Fig. 3F). Surprisingly, the Δ*terC* and CΔ*terC* strains always exhibited similar growth patterns to the WT strain when Mn concentrations gradually increased (Fig. 3B through F). Moreover, when other metals were added, all mutants exhibited similar growth to the WT strain (Fig. S2).

Taken together, these results indicate that MneA, rather than TerC, plays a primary role in *V. parahaemolyticus* resistance to Mn stress.

### TerC facilitates *V. parahaemolyticus* growth under high Mn concentrations in weakly acidic TSB (pH 6.8)

**Fig 4:**
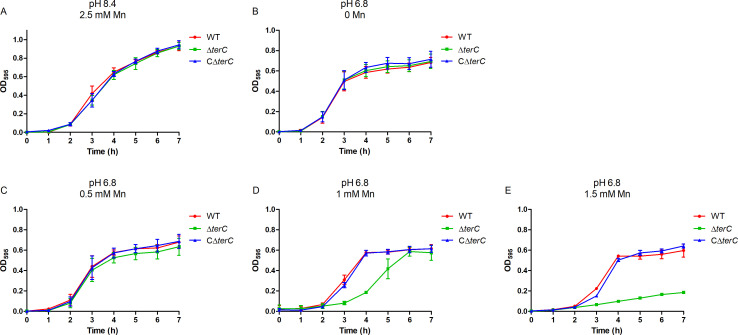
The effect of added Mn on the growth of the WT, Δ*terC*, and CΔ*terC* strains in TSB (pH 8.4) or TSB (pH 6.8). (**A**) Growth curves of the strains in TSB (pH 8.4) supplemented with 2.5 mM Mn. (**B–E**) The strains were grown in TSB (pH 6.8) supplemented with 0 mM (**B**), 0.5 mM (**C**), 1 mM (**D**), or 1.5 mM (**E**) Mn. For the complemented strains, chloramphenicol (25 μg/mL) and IPTG (1 mM) were added to the medium to maintain the plasmid and induce gene expression. The WT and mutant strains were grown without chloramphenicol and IPTG. Please note that all pH values reported refer to the initial pH at inoculation; in our experiments, the medium pH dropped by approximately 1 pH unit after 7 h of culture. The results represent the means and standard deviations from three independent experiments performed in duplicate.

In *E. coli*, Alx, a homolog of TerC, precisely tunes intracellular Mn concentration by Mn efflux; its activity is highest during alkaline stress (27). Therefore, we examined the role of *terC* in *V. parahaemolyticus* growth under Mn stress in alkaline TSB (pH 8.4). Surprisingly, under alkaline conditions, even if the Mn concentration was increased to 2.5 mM, the growth of Δ*terC* did not differ from that of the WT and CΔ*terC* strains (Fig. 4A). Next, we evaluated the growth of the WT, Δ*terC*, and CΔ*terC* strains in TSB (pH 6.8) supplemented with various Mn concentrations. In TSB (pH 6.8) without Mn supplementation, all strains entered stationary phase earlier than in standard TSB and showed similar growth (Fig. 4B). When 0.5 mM Mn was added, the growth of the three strains still did not differ obviously (Fig. 4C). However, in the presence of 1 mM Mn, Δ*terC* showed moderate growth impairment compared with the WT and CΔ*terC* strains (Fig. 4D). Moreover, when the Mn concentration was increased to 1.5 mM, Δ*terC* showed severe growth impairment, while CΔ*terC* exhibited growth similar to the WT strain (Fig. 4E).

**Fig 5:**
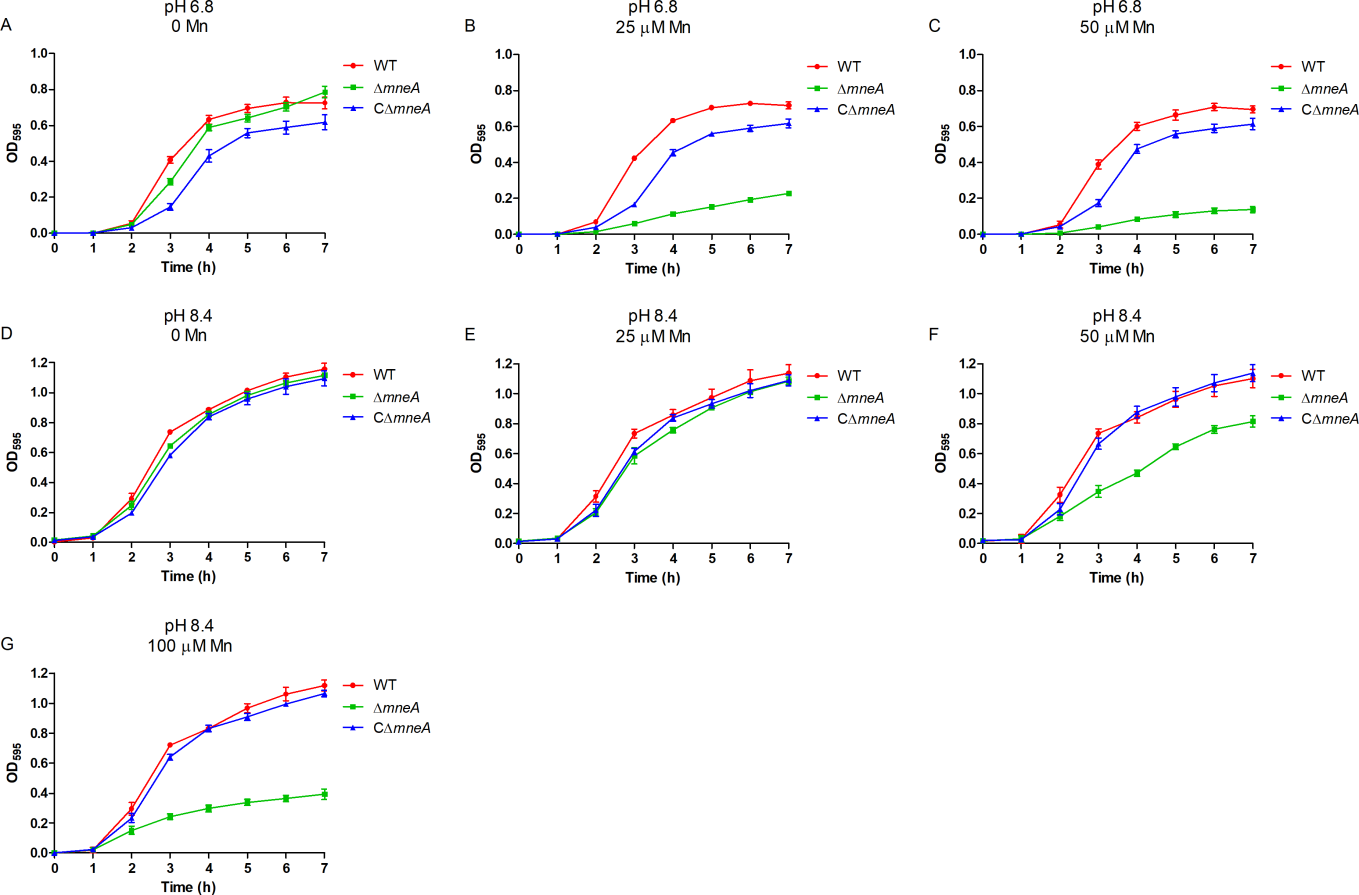
The effect of added Mn on the growth of the WT, Δ*mneA*, and CΔ*mneA* strains in TSB (pH 6.8) or TSB (pH 8.4). (**A–C**) Growth curves of the strains in TSB (pH 6.8) supplemented with 0 μM (**A**), 25 μM (**B**), or 50 μM (**C**) Mn. (**D–G**) The strains were grown in TSB (pH 8.4) supplemented with 0 μM (**D**), 25 μM (**E**), 50 μM Mn (**F**), or 100 μM (**G**) Mn. For the complemented strains, chloramphenicol (25 μg/mL) and IPTG (1 mM) were added to the medium to maintain the plasmid and induce gene expression. The WT and mutant strains were grown without chloramphenicol and IPTG. Please note that all pH values reported refer to the initial pH at inoculation; in our experiments, the medium pH dropped by approximately 1 pH unit after 7 h of culture. The results represent the means and standard deviations from three independent experiments performed in duplicate.

We also examined the role of *mneA* in *V. parahaemolyticus* growth under Mn stress at pH 6.8 and pH 8.4. In TSB (pH 6.8), the WT and Δ*mneA* strains exhibited similar growth in the absence of Mn supplementation (Fig. 5A), whereas Δ*mneA* showed growth impairment when elevated concentrations of Mn were added (Fig. 5B and C). Complementation of *mneA* could partly restore the mutant’s growth (Fig. 5A through C). In TSB (pH 8.4) without Mn supplementation or supplemented with 25 μM Mn, the WT, Δ*mneA*, and CΔ*mneA* strains exhibited similar growth (Fig. 5D and E). However, in the presence of 50 or 100 μM Mn, Δ*mneA* showed remarkable growth inhibition, while CΔ*mneA* exhibited growth similar to the WT strain (Fig. 5F and G).

Taken together, TerC facilitates *V. parahaemolyticus* growth under high Mn conditions in weakly acidic medium, while MneA functions in both weakly acidic and alkaline media.

### MneA and TerC protect *V. parahaemolyticus* against Mn-induced bacteriostasis

**Fig 6:**
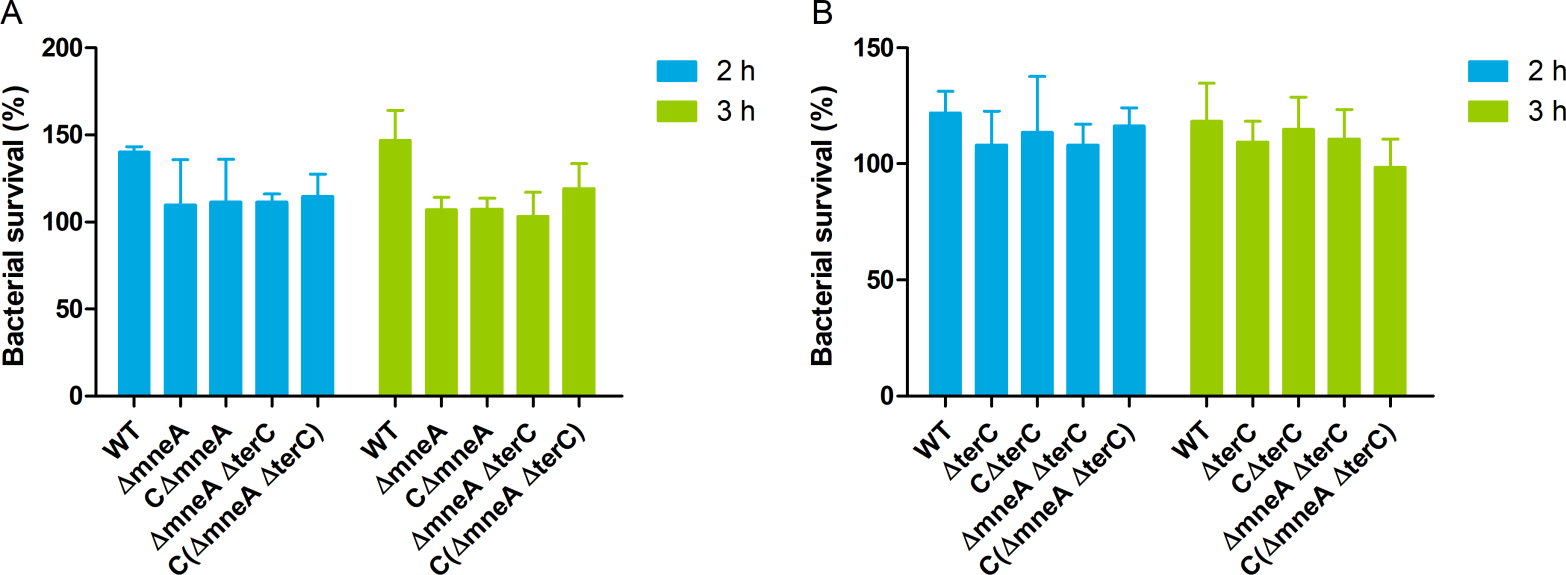
Bacterial survival assay showing the bacteriostatic effect of Mn on the mutant strains. (**A**) The WT, Δ*mneA*, CΔ*mneA*, Δ*mneA* Δ*terC*, and C(Δ*mneA* Δ*terC*) strains were treated with 2 mM Mn in standard TSB for 3 h. (**B**) The WT, Δ*terC*, CΔ*terC*, Δ*mneA* Δ*terC*, and C(Δ*mneA* Δ*terC*) strains were treated with 2 mM Mn in TSB (pH 6.8) for 3 h. For the complemented strains, chloramphenicol (25 μg/mL) and IPTG (1 mM) were added to the medium to maintain the plasmid and induce gene expression. The WT and mutant strains were grown without chloramphenicol and IPTG. Aliquots were taken at 0, 2, and 3 h, serially diluted, and plated on TSA to enumerate viable bacteria. The survival rate was calculated by normalizing the bacterial concentration at each time point to that at 0 h. Please note that all pH values reported refer to the initial pH at inoculation; in our experiments, the medium pH dropped by approximately 1 pH unit after 7 h of culture. The results represent the means and standard deviations from three independent experiments.

In order to further clarify the role of *mneA* and *terC* in *V. parahaemolyticus* resistance to Mn stress, all the strains were cultured until the mid-exponential phase. Then, we treated the WT, Δ*mneA*, CΔ*mneA*, Δ*mneA* Δ*terC*, and C(Δ*mneA* Δ*terC*) strains with 2 mM Mn in standard TSB for 3 h, and analyzed their survival at 2 and 3 h. Similarly, the WT, Δ*terC*, CΔ*terC*, Δ*mneA* Δ*terC*, and C(Δ*mneA* Δ*terC*) strains were treated with 2 mM Mn in TSB (pH 6.8) for 3 h, and analyzed for bacterial survival. As shown in Fig. 6A, following treatment with 2 mM Mn for 2 and 3 h, the survival rates of Δ*mneA* and Δ*mneA* Δ*terC* remained above 100%. Additionally, neither Δ*terC* nor Δ*mneA* Δ*terC* exhibited reduced survival after 2 or 3 h of Mn treatment (Fig. 6B).

These data, together with the growth defect of the mutants under Mn stress, indicate that both MneA and TerC protect *V. parahaemolyticus* against the bacteriostatic effect of Mn.

### The deletion of *mneA* or *terC* significantly increases intracellular Mn content

**Fig 7:**
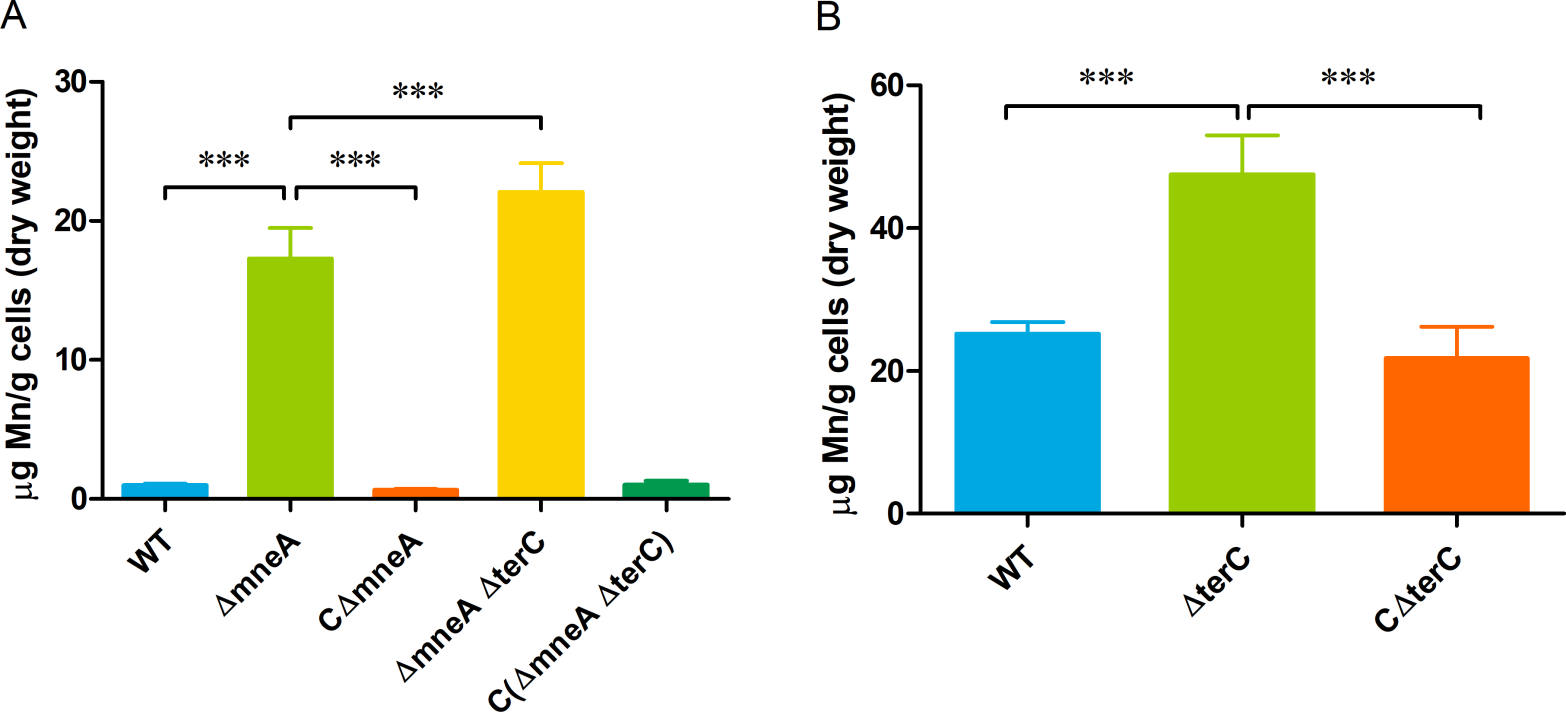
ICP-MS analysis of intracellular Mn content in the *V. parahaemolyticus* strains. (**A**) The intracellular Mn content in the WT, Δ*mneA*, CΔ*mneA*, Δ*mneA* Δ*terC*, and C(Δ*mneA* Δ*terC*) strains cultured in standard TSB supplemented with 25 μM Mn. (**B**) The intracellular Mn content in the WT, Δ*terC*, and CΔ*terC* strains cultured in TSB (pH 6.8) supplemented with 0.5 mM Mn. For the complemented strains, chloramphenicol (25 μg/mL) and IPTG (1 mM) were added to the medium to maintain the plasmid and induce gene expression. The WT and mutant strains were grown without chloramphenicol and IPTG. Please note that all pH values reported refer to the initial pH at inoculation; in our experiments, the medium pH dropped by approximately 1 pH unit after 7 h of culture. The results represent the means and standard deviations from six independent prepared samples. The data were analyzed using one-way analysis of variance along with Bonferroni’s post-test. ***, *P*  <  0.001.

To elucidate the molecular mechanism by which *mneA* and *terC* mediate the response of *V. parahaemolyticus* to Mn bacteriostatic activity, we performed inductively coupled plasma-mass spectrometry (ICP-MS) to analyze the levels of Mn in the cells grown in the presence of Mn. When cultured in TSB (pH 7.3) supplemented with 25 μM Mn, intracellular accumulation of Mn in Δ*mneA* was considerably higher than that in the WT and CΔ*mneA* strains, reaching 17.31 ± 2.19 µg Mn per g cells (dry weight) (Fig. 7A). On the other hand, Mn accumulation in CΔ*mneA* was equivalent to that in the WT strain, reaching only less than 1 µg Mn per g cells (dry weight) (Fig. 7A). Furthermore, Δ*mneA* Δ*terC* accumulated 22.06 ± 2.09 µg Mn per g cells (dry weight), significantly higher than Δ*mneA* did (Fig. 7A). After growth in TSB (pH 6.8) supplemented with 0.5 mM Mn, the WT strain accumulated 25.21 ± 1.69 µg Mn per g cells (dry weight). When *terC* was deleted, Mn accumulation was nearly twice that noted in the WT strain; however, the intracellular Mn content in CΔ*terC* was comparable to that in the WT strain (Fig. 7B). Additionally, while the single mutants (either Δ*mneA* or Δ*terC*) accumulated Zn content comparable to the WT strain, Δ*mneA* Δ*terC* accumulated significantly higher Zn content (55.58 ± 7.20 µg Zn per g cells [dry weight]) than both the WT strain (44.40 ± 4.88 µg Zn per g cells [dry weight]) and C(Δ*mneA* Δ*terC*) (41.92 ± 2.63 µg Zn per g cells [dry weight]) (Fig. S3). Collectively, the deletion of *mneA* or *terC* can significantly increase intracellular Mn accumulation, indicating that MneA and TerC confer *V. parahaemolyticus* resistance to Mn bacteriostatic activity by Mn efflux.

### MneA contributes to the virulence of *V. parahaemolyticus* in zebrafish

**Fig 8:**
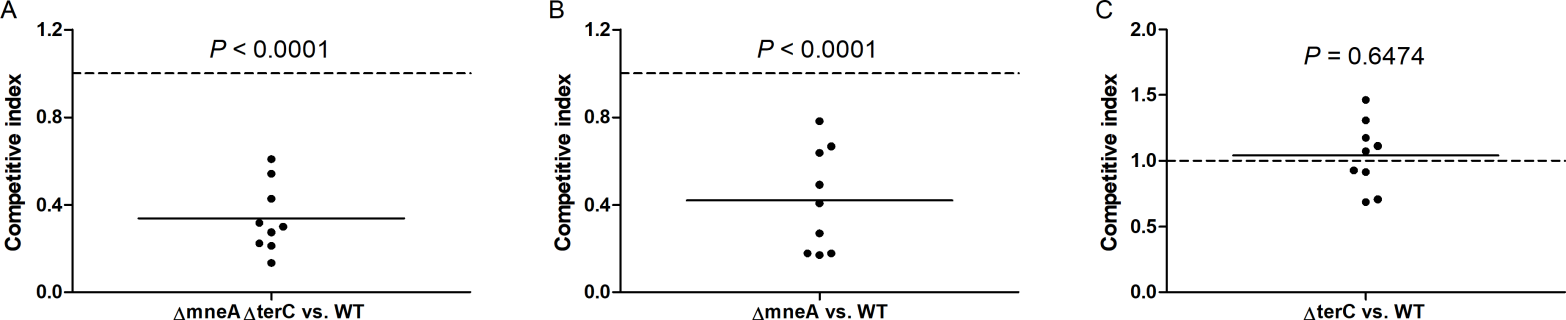
Competitive index (CI) of Δ*mneA* Δ*terC* (**A**), Δ*mneA* (**B**), and Δ*terC* (**C**) against the WT strain in the intestine of zebrafish. A 1:1 mixture of each mutant and the WT strain was intraperitoneally injected into zebrafish. At 24 h after infection, the full intestines were collected, and bacteria recovered from the samples were identified by colony PCR. The CI was calculated as the ratio of the mutant to the WT strain in each zebrafish divided by that in the inoculum. The CI values were compared to 1 (equal competitiveness) using the two-tailed paired *t*-test to determine significance.

To assess the role of Mn detoxification in the pathogenicity of *V. parahaemolyticus*, we performed a competitive infection assay using a zebrafish model. The WT strain was mixed with each mutant at a ratio of 1:1, and then injected into the abdominal cavity of zebrafish. After 24 h, bacteria were recovered from the fish intestine and identified by colony PCR to evaluate the ratio of each mutant to the WT strain. As shown in Fig. 8A, the competitive index (CI) values of Δ*mneA* Δ*terC* against the WT strain were 0.3373 ± 0.1576, significantly lower than 1 (equal competitiveness), indicating that the virulence of the double mutant was significantly reduced compared with the WT strain. Moreover, the CI values of Δ*mneA* against the WT strain were 0.4194 ± 0.2358, significantly lower than 1 (Fig. 8B). Nevertheless, the CI values of Δ*terC* against the WT strain were 1.041 ± 0.2603, which were not significantly different from 1 (Fig. 8C). Taken together, MneA, rather than TerC, contributes to the virulence of *V. parahaemolyticus* in zebrafish.

### The transcriptome of *V. parahaemolyticus* is altered in response to Mn bacteriostatic activity

**Fig 9:**
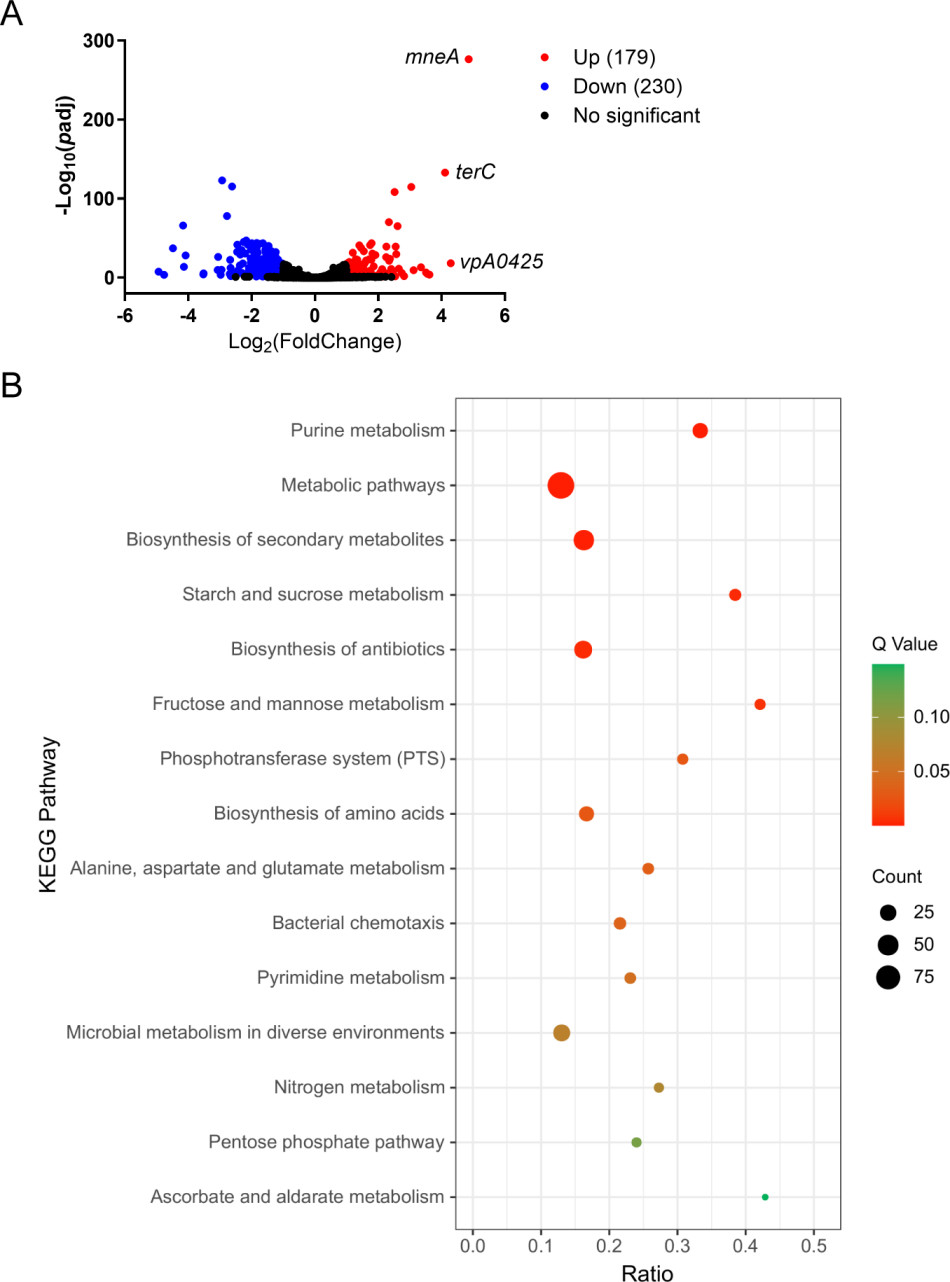
Transcriptional response of *V. parahaemolyticus* to Mn bacteriostatic activity. (**A**) The transcriptome changes of *V. parahaemolyticus* following treatment with 1 mM Mn compared to H_2_O. (**B**) The top 15 enriched KEGG pathways of the DEGs. The KEGG pathways with a *q* value < 0.05 were considered significantly enriched.

In order to reveal the molecular mechanism by which *V. parahaemolyticus* responds to excessive Mn, we extracted the total RNA of RIMD2210633 treated with H_2_O and 1 mM Mn, and performed transcriptome analysis. Under Mn stress, a total of 409 genes, accounting for approximately 8.6% of the total genome, were significantly differentially expressed. Of these, 179 genes were upregulated and 230 genes were downregulated. *mneA* and *terC* were among the most significantly upregulated genes (Fig. 9A; Table S1). Additionally, RNA sequencing reads mapped across the entire coding regions of *mneA* and *terC* under both control and Mn-supplemented conditions; no abrupt drop-off in coverage was observed at or immediately downstream of the predicted riboswitch region (Fig. S4). The presence of full-length transcripts under both conditions is consistent with multiple regulatory scenarios, including alleviation of riboswitch-mediated transcriptional termination, increased transcription initiation, or enhanced transcript stability. Given that overall read coverage increased substantially under Mn stress without a corresponding change in the coverage profile across the riboswitch region, the precise contribution of each mechanism remains to be determined. Furthermore, *VP1164* (annotated as a putative Mn transporter) and *VP1383* were upregulated by approximately 12- and 3-fold, respectively (Table S1). Several genes encoding hypothetical proteins were also significantly differentially expressed (Table S1). The differentially expressed genes could be enriched into 68 KEGG pathways; the top 15 enriched pathways are shown in Fig. 9B. Among them, 11 pathways were significantly enriched, including purine metabolism, metabolic pathways, biosynthesis of secondary metabolites, starch and sucrose metabolism, biosynthesis of antibiotics, fructose and mannose metabolism, phosphotransferase system, biosynthesis of amino acids, alanine, aspartate and glutamate metabolism, bacterial chemotaxis, and pyrimidine metabolism. These results suggest that *V. parahaemolyticus* modulates the expression of multiple genes in response to Mn bacteriostatic activity.

## DISCUSSION

While Mn plays a crucial role in the physiology and pathogenicity of bacteria, it can be toxic in excessive amounts. Metal poisoning is a nutritional immunity strategy employed by vertebrates to defend against pathogen invasion (34). Correspondingly, bacteria have evolved various mechanisms, such as different types of Mn efflux systems, to maintain intracellular Mn homeostasis. In recent years, Mn efflux systems have been characterized in numerous bacteria. Some of these systems have been demonstrated to be associated with bacterial oxidative stress resistance and virulence (22, 35). Nevertheless, such Mn efflux systems and their mediated functions have rarely been reported in *Vibrio* species. In this study, we identified two genes, *mneA* and *terC*, from the *V. parahaemolyticus* genome. Our results revealed that these two genes tune intracellular Mn homeostasis by Mn efflux. Among them, *terC* functions in weakly acidic environment. Additionally, *mneA* contributes to the virulence of *V. parahaemolyticus*.

BLASTP analysis revealed that MneA in *V. parahaemolyticus* exhibits high homology with MneA in *V. cholerae*. In contrast, TerC exhibits relatively low homology with related proteins from other bacteria; however, this finding may be attributed to the distant relationship between *V. parahaemolyticus* and these bacteria. Additionally, genes homologous to *mneA* and *terC* are widely present in other *Vibrio* species. Consequently, investigating the functions of *mneA* and *terC* in *V. parahaemolyticus* holds great significance for elucidating the molecular mechanism by which *Vibrio* species maintain Mn homeostasis.

Under excessive metal stress, bacteria typically regulate the expression of specific genes (36). Hence, we employed qRT-PCR to assess the expression of *mneA* and *terC* under diverse metal stress conditions. Intriguingly, the expression levels of *mneA* and *terC* were significantly upregulated only upon treatment with Mn. Subsequent transcriptome sequencing also verified this result. Growth curve analyses indicated that the Δ*mneA* mutant was highly sensitive to Mn in standard, alkaline, and weakly acidic media. Notably, when 25 μM Mn was added to standard or weakly acidic medium, the Δ*mneA* mutant exhibited distinct growth impairments. As the Mn concentrations increased, the inhibitory effect became more pronounced. As expected, ICP-MS analysis revealed that Δ*mneA* accumulated much higher Mn than the WT and complemented strains when they were grown in the presence of Mn. These findings indicate that *mneA* maintains Mn homeostasis in *V. parahaemolyticus* by mediating Mn efflux. Consistent with this, the Δ*mneA* mutant in *V. cholerae* also exhibited extreme sensitivity to Mn, while remaining unaffected by other metals (25). MneA exhibits a high degree of conservation across *Vibrio* species, and its characteristics are remarkably similar in *V. parahaemolyticus* and *V. cholerae*. Our findings provide new evidence for the widespread presence of MneA as a Mn exporter in *Vibrio* species.

The role of TerC family proteins in maintaining Mn homeostasis within bacteria remains controversial. An early study has proposed that the TerC family protein Alx in *E. coli* increases the intracellular Mn concentration, implicating its involvement in Mn uptake (37). However, more recent findings have revealed that Alx mediates Mn efflux under alkaline conditions, safeguarding cells against Mn toxicity (27). Moreover, the TerC family proteins in *R. anatipestifer* (28) and *B. subtilis* (29) have been demonstrated to be Mn exporters. Notably, in *B. subtilis*, two TerC family proteins (MeeF and MeeY) participate in efflux and aid in metalating extracellular Mn-dependent enzymes (30). Our data indicate that *V. parahaemolyticus* TerC mediates Mn efflux in a weakly acidic environment. In standard or alkaline medium, the Δ*terC* mutant did not exhibit growth inhibition even upon the addition of high concentrations of Mn. However, in a weakly acidic medium, Δ*terC* started to exhibit moderate growth retardation when 1 mM Mn was added. As the Mn concentration was increased further, the strain exhibited severe growth impairments. ICP-MS analysis also revealed that the Δ*terC* mutant accumulated nearly twice as much Mn as the WT and complemented strains. Furthermore, in standard medium supplemented with 25 μM Mn, the Δ*mneA* Δ*terC* double mutant was more sensitive to Mn stress and accumulated significantly higher Mn content than Δ*mneA*, indicating that TerC contributes to Mn resistance in *V. parahaemolyticus* under conditions where MneA is absent. Collectively, these findings support the role of TerC in Mn efflux under weakly acidic conditions. Medium acidification during bacterial growth is a common phenomenon (38–40). Accordingly, in our experiments, we observed that the pH of the growth medium decreased by approximately 1 pH unit over the cultivation period. Thus, even when starting from a standard or alkaline medium, the culture may become weakly acidic at later stages. This raises the possibility that TerC may exert its Mn efflux function at later stages, even when the medium was initially standard or alkaline. It is also plausible that TerC may have additional roles in metalloenzyme metalation or other post-efflux cellular processes, similar to its homolog in *B. subtilis* (30). Redundant Mn homeostasis mechanisms in *V. parahaemolyticus* could enhance the adaptability of this bacterium to complex environments.

Mn detoxification plays an important role in the pathogenesis of many pathogens (41). In previous studies, some Mn efflux-related proteins have been confirmed to be closely related to bacterial virulence. For instance, MntE of *Streptococcus suis* (35) and *Staphylococcus aureus* (42), MntX of *Neisseria meningitidis* (43), and EmfA of *Brucella abortus* (44), have been confirmed to affect bacterial virulence and survival. Notably, not all Mn exporters are linked to virulence. For instance, the Mn efflux genes *metA* and *metB* in *R. anatipestifer* are not involved in its virulence (28). Zebrafish serve as an ideal model for assessing the virulence of *V. parahaemolyticus* (45, 46), and competitive infection experiments have been extensively employed to assess bacterial virulence (47, 48). Therefore, in this study, we performed a competitive infection experiment using a zebrafish model to compare the virulence of the WT strain with that of each mutant. Our results revealed that the deletion of *mneA* significantly decreased the virulence of *V. parahaemolyticus*, while the deletion of *terC* had no notable impact.

The expression of genes encoding metal transporters is typically controlled by metal sensors (49). Mn transporters in bacteria are mainly regulated by transcription factors MntR/Mur or riboswitch *yybP-ykoY* (50). The *yybP-ykoY* motif represents one of the most extensively distributed and abundant bacterial riboswitches, exhibiting remarkable sensitivity to Mn (51). Genes regulated by *yybP-ykoY* play a crucial role in the regulation of Mn homeostasis across numerous bacteria, including *S. pneumoniae* (52), *E. coli* (53), *B. subtilis* (21), and *Lactococcus lactis* (51). Notably, the Mn export protein identified in *V. cholerae* is also predicted to be under the control of its upstream *yybP-ykoY* riboswitch (25). In this study, we used RegPrecise and predicted that the Mn exporters MneA and TerC in *V. parahaemolyticus* are also regulated by its upstream *yybP-ykoY* riboswitch. Generally, the *yybP-ykoY* riboswitch modulates transcription termination or translation initiation processes in response to Mn levels (21). However, our RNA sequencing data showed that, under both control and Mn-supplemented conditions, read coverage was distributed across the entire coding regions of *mneA* and *terC* without a pronounced drop-off at the predicted riboswitch region. This observation suggests that the riboswitch may not primarily function through transcriptional termination in *V. parahaemolyticus* under the conditions tested, and that the upregulation of *mneA* and *terC* under Mn stress could involve other regulatory mechanisms, such as enhanced transcription initiation or transcript stabilization. Further studies are needed to elucidate the specific regulatory mechanism in *V. parahaemolyticus*.

In the *V. parahaemolyticus* RIMD2210633 genome, the *VP1164* gene is annotated as a putative Mn transporter. VP1164 shares amino acid sequence homology with the novel MntX transporters, including *Roseovarius nubinhibens* ISM_02005 (40.94% identity), *Candidatus Pelagibacter* SAR11G3_00277 (35.84% identity), and *V. cholerae* VC_1688 (62.96% identity). These proteins are involved in Mn import (54). Based on homology, VP1164 should be characterized as a Mn importer. However, our RNA sequencing data revealed that *VP1164* was upregulated by approximately 12-fold under Mn-supplemented conditions. Since Mn importers are typically repressed rather than induced under Mn excess, this upregulation raises the possibility that VP1164 may function as a Mn exporter, or alternatively, may have a more complex role in Mn homeostasis that differs from its characterized homologs. Additionally, *VP1164* was significantly induced under Cu excess (27.88-fold) (55) and Zn limitation (32.71-fold) (56, 57), indicating that it might have broader roles in metal homeostasis. Another gene of interest is *VP1383*, which is annotated as a TerC family protein. This gene was moderately upregulated (~3-fold) under Mn-supplemented conditions. VP1383 shares amino acid sequence identity (approximately 23–26%) with Alx from *E. coli* and YceF/YkoY from *B. subtilis*, which have been characterized as Mn exporters (27, 29). Its induction under Mn excess is consistent with a potential role in Mn efflux, although functional validation is needed. Furthermore, several genes encoding hypothetical proteins were significantly differentially expressed under Mn-supplemented conditions. While the exact functions of these proteins remain unknown, their strong induction or repression under Mn stress suggests that they may play important roles in Mn homeostasis or general metal stress responses. The roles of VP1164, VP1383, and these hypothetical proteins in metal homeostasis and virulence warrant further exploration in *V. parahaemolyticus*.

In summary, the Mn exporters MneA and TerC promote *V. parahaemolyticus* growth under distinct Mn conditions. They can protect this bacterium against Mn-induced bacteriostasis by mediating Mn efflux. Notably, MneA contributes to the virulence of *V. parahaemolyticus* in zebrafish, whereas TerC has no such effect.

## MATERIALS AND METHODS

### Bacterial strains, plasmids, and growth conditions

The bacterial strains and plasmids utilized in this study are presented in Table S2, and the primer sequences are provided in Table S3. *E. coli* was grown at 37°C in Luria–Bertani (LB) broth or on LB agar. For the construction of mutants and complemented strains, *V. parahaemolyticus* RIMD2210633 and its derivative strains were cultured in LB, while TSB or tryptic soy agar (TSA) was employed for the other assays. Unless specified otherwise, standard TSB (pH 7.3) or TSA was used. Please note that all pH values reported refer to the initial pH at inoculation; in our experiments, the medium pH dropped by approximately 1 pH unit after 7 h of culture. When appropriate, the following antibiotics were added at the indicated concentrations: carbenicillin (50 μg/mL) and chloramphenicol (25 μg/mL).

### RNA extraction, qRT-PCR, and RNA sequencing

Overnight cultured RIMD2210633 was transferred into fresh TSB at a ratio of 1:100 and cultured until the early exponential phase (OD_600_ = 0.6–0.8). Seven 1 mL aliquots of the bacterial solution were then collected. Six aliquots were treated with different metals (1 mM FeSO_4_, 1 mM MnSO_4_, 0.5 mM ZnSO_4_, 1 mM CuSO_4_, 0.5 mM CoSO_4_, and 1 mM NiSO_4_) and one was treated with H_2_O. After incubating at 37°C for 15 min, bacterial cells were collected to extract total RNA using an Eastep Super Total RNA Isolation Kit (Promega). In order to guarantee the accuracy of the experimental data, samples were obtained from three independent experiments. The integrity of the RNA samples was verified by agarose gel electrophoresis, and their concentrations were measured using a NanoDrop 200 spectrophotometer.

The qualified total RNA samples extracted from RIMD2210633 on treatment with various metals and H_2_O were utilized as templates to synthesize cDNA using HisScript II QRT SuperMix for qPCR (+gDNA wiper; Vazyme). qRT-PCR was performed on the StepOnePlus Real-Time PCR System (Applied Biosystems) using ChamQ SYBR qPCR Master Mix (Vazyme) and specific primers (Table S3). Tenfold-diluted cDNA served as a template for this reaction, and the *gyrB* gene served as an internal control. Relative gene expression was calculated using the 2^−ΔΔCT^ method (58).

Additionally, overnight cultured RIMD2210633 was transferred into fresh TSB at a ratio of 1:100 and cultured until the early exponential phase. Then, two 1 mL aliquots were taken: one was treated with 1 mM MnSO_4_ and the other with H_2_O. After incubating at 37°C for 15 min, bacterial cells were harvested by centrifugation. Subsequently, RNA was extracted using an Eastep Super Total RNA Isolation Kit (Promega). The integrity of the RNA samples was verified by agarose gel electrophoresis, and their concentrations were measured using a NanoDrop 200 spectrophotometer. The qualified RNA samples were sent to Azenta Life Sciences for RNA sequencing.

### Construction of mutant and complemented strains

The Δ*mneA* and Δ*terC* mutants lacking *mneA* and *terC*, respectively, were generated through allelic exchange by using the pDM4 plasmid (59), as described previously (60). For the construction of the double mutant Δ*mneA* Δ*terC* lacking both *mneA* and *terC*, the pDM4-Δ*mneA* plasmid was transferred into Δ*terC*.

The complemented strains CΔ*mneA* and CΔ*terC* were generated using the pMMB207 plasmid (61). Briefly, the target gene and an additional ribosome-binding site were cloned into pMMB207. The resulting plasmid was transformed into *E. coli* S17-1 *λpir* and then conjugated into the corresponding mutant. For generating the complemented strain C(Δ*mneA* Δ*terC*), *mneA* complementation was generated following the procedure for mutant construction, while *terC* complementation was generated using pMMB207. In brief, *mneA* and its flanking regions were amplified and cloned into pDM4, generating pDM4-C*mneA*. This plasmid was transformed into *E. coli* S17-1 *λpir* and then conjugated into Δ*mneA* Δ*terC*. Transconjugants were selected on LB agar supplemented with carbenicillin and chloramphenicol, cultured in LB broth with 15% sucrose, and then streaked onto LB agar containing 15% sucrose. Sucrose-resistant colonies were identified as candidate intermediate strains (designated as Δ*mneA* Δ*terC::mneA; mneA*-complemented) and verified by PCR. Subsequently, pMMB207-*terC* was introduced into the intermediate strain for *terC* complementation. For complementation strains, isopropyl β-d-1-thiogalactopyranoside (IPTG) was added to a final concentration of 1 mM to induce gene expression.

All the constructed strains were verified by PCR (Fig. S5A through F) and DNA sequencing.

### Bacterial growth in standard TSB

Overnight cultured WT, Δ*mneA*, Δ*terC*, Δ*mneA* Δ*terC*, CΔ*mneA*, CΔ*terC*, and C(Δ*mneA* Δ*terC*) strains were transferred into fresh TSB at a ratio of 1:100 and cultured until the mid-exponential phase (OD_600_ = 1.5–2). Then, these strains were diluted in fresh TSB supplemented with different concentrations of MnSO_4_ (0, 0.025, 0.05, 0.1, 0.5, and 1 mM) or excessive concentrations of other metals (2 mM FeSO_4_, 2 mM CuSO_4_, 2 mM ZnSO_4_, 0.25 mM CoSO_4_, and 0.75 mM NiSO_4_) at a ratio of 1:100. In the medium containing FeSO_4_, trisodium citrate dihydrate (TCD, 1 g/L) was added to alleviate Fe(II) precipitation (62). The cultures were inoculated in 96-well plates (200 μL per well, two replicates per condition) and cultured at 37°C, 120 rpm for 7 h. The OD_595_ was measured hourly using a CMax Plus microplate reader (Molecular Devices).

### Bacterial growth in alkaline or weakly acidic TSB

The pH of TSB was adjusted to 6.8 and 8.4 using HCl and NaOH, respectively. Overnight cultured WT, Δ*terC*, and CΔ*terC* strains were transferred into fresh TSB at a ratio of 1:100, cultured until the mid-exponential phase, and then diluted in TSB (pH 8.4) supplemented with 2.5 mM MnSO_4_ at a ratio of 1:100. The cultures were inoculated in 96-well plates (200 μL per well, two replicates per condition) and cultured at 37°C, 120 rpm. The OD_595_ was measured hourly using a CMax Plus microplate reader (Molecular Devices).

In another experiment, overnight cultured WT, Δ*mneA*, CΔ*mneA*, Δ*terC*, and CΔ*terC* strains were transferred into fresh TSB at a ratio of 1:100 and cultured until the mid-exponential phase. The WT, Δ*mneA*, and CΔ*mneA* strains were diluted 1:100 in TSB (pH 6.8) or TSB (pH 8.4) supplemented with MnSO_4_ concentrations of 0, 25, 50, and 100 μM, while the WT, Δ*terC*, and CΔ*terC* strains were diluted 1:100 in TSB (pH 6.8) supplemented with MnSO_4_ concentrations of 0, 0.5, 1, and 1.5 mM. The cultures were inoculated in 96-well plates (200 μL per well, two replicates per condition) and cultured at 37°C, 120 rpm. The OD_595_ was measured hourly using a CMax Plus microplate reader (Molecular Devices).

### Bacterial survival assays

Overnight cultured WT, Δ*mneA*, Δ*terC*, Δ*mneA* Δ*terC*, CΔ*mneA*, CΔ*terC*, and C(Δ*mneA* Δ*terC*) strains were transferred into fresh TSB at a ratio of 1:100 and cultured until the mid-exponential phase (OD_600_ = 1.5–2). Then, the WT, Δ*mneA*, CΔ*mneA*, Δ*mneA* Δ*terC*, and C(Δ*mneA* Δ*terC*) strains were harvested and adjusted to 1 × 10^9^ CFU/mL with standard TSB. Additionally, the WT, Δ*terC*, CΔ*terC*, Δ*mneA* Δ*terC*, and C(Δ*mneA* Δ*terC*) strains were harvested and adjusted to 1 × 10^9^ CFU/mL with TSB (pH 6.8). From each suspension, 1 mL aliquots were taken, treated with 2 mM MnSO_4_, and incubated at 37°C for 3 h. At 0, 2, and 3 h, 100 μL aliquots were taken, serially diluted, and plated on TSA to enumerate viable bacteria. The bacterial survival rate was calculated by normalizing the bacterial concentration at each time point to that at 0 h.

### ICP-MS analysis

The WT, Δ*mneA*, CΔ*mneA*, Δ*mneA* Δ*terC*, C(Δ*mneA* Δ*terC*), Δ*terC*, and CΔ*terC* strains were grown in standard TSB until the mid-exponential phase. Then, the WT, Δ*mneA*, CΔ*mneA*, Δ*mneA* Δ*terC*, and C(Δ*mneA* Δ*terC*) strains were diluted 1:100 in standard TSB supplemented with 25 μM MnSO_4_. Additionally, the WT, Δ*terC*, and CΔ*terC* strains were diluted 1:100 in TSB (pH 6.8) supplemented with 0.5 mM MnSO_4_. All cultures were incubated at 37°C, 220 rpm for 6 h. Bacterial cells were collected, washed, and dried as described previously (56). Following this, the samples were weighed, nitrated with 66% nitric acid at 70°C for 48 h, and diluted with metal-free water to a final concentration of 2% nitric acid. The Mn/Zn content, defined as micrograms of Mn/Zn per gram of cells (dry weight), was measured using PerkinElmer Elan DRC-e at Yangzhou University, according to the manufacturer’s instructions.

### Competitive infection assay using a zebrafish model

The WT, Δ*mneA*, Δ*terC*, Δ*mneA* Δ*terC* strains were grown in TSB at 30°C, 220 rpm for 12 h. Then, 1 mL of each culture was centrifuged to collect bacteria, and the bacterial concentration was adjusted to 1 × 10^9^ CFU/mL with PBS. Subsequently, each mutant was mixed with the WT strain at a ratio of 1:1. Twenty-seven zebrafish (3–4 months old) were anesthetized with 0.1% ethyl 3-aminobenzoate methane sulfonate (Sigma-Aldrich) and intraperitoneally injected with 10 μL of the mixed bacteria (nine zebrafish per mixture). At 24 h post-infection, the zebrafish were sacrificed, and the full intestines were homogenized in PBS, and diluted. Then, 100 μL of the diluted sample was coated on thiosulfate citrate bile-salts sucrose agar (TCBS) and cultured overnight at 37°C. A total of 90–100 single colonies were randomly selected from each sample, and the proportion of mutant:WT in the sample was determined by colony PCR. The CI was calculated as the ratio of the mutant to the WT strain in each zebrafish, divided by that in the inoculum.

### Bioinformatic and statistical analyses

RegPrecise 3.2 (31) was used to predict the regulons of the riboswitch *yybP-ykoY* in *V. parahaemolyticus* RIMD2210633 and other species in *Vibrionaceae*. KEGG pathway enrichment analysis was performed using KOBAS-i (63), and visualized using Hiplot Pro (https://hiplot.com.cn/home/index.html).

Statistical analyses were performed using GraphPad Prism 5. Gene expression and Mn content were analyzed using one-way analysis of variance along with Bonferroni’s post-test. A two-tailed paired *t*-test was used to compare the CI value with 1 (a value indicating equal competitiveness).

## Data Availability

RNA sequencing data have been submitted to Gene Expression Omnibus (GEO) under accession number GSE287416. Data will be made available on request.

## SUPPLEMENTAL MATERIAL

Supplemental material aem.01754-25-s0001.docx Fig. S1 to S5; Tables S2 and S3.

Table S1 aem.01754-25-s0002.xlsx Summary of differentially expressed genes in *V. parahaemolyticus* following Mn treatment.

ASM does not own the copyrights to Supplemental Material that may be linked to, or accessed through, an article. The authors have granted ASM a non-exclusive, world-wide license to publish the Supplemental Material files. Please contact the corresponding author directly for reuse.
