# Supplementary material for: MneA and TerC exhibit distinct roles in manganese detoxification and virulence in Vibrio parahaemolyticus
Source: Appl Environ Microbiol. 2026 May 20;92(6):e01754-25. doi: 10.1128/aem.01754-25 (PMC13274385; doi:10.1128/aem.01754-25)
Supplement: Supplemental material — Fig. S1 to S5; Tables S2 and S3. [file aem.01754-25-s0001.docx]

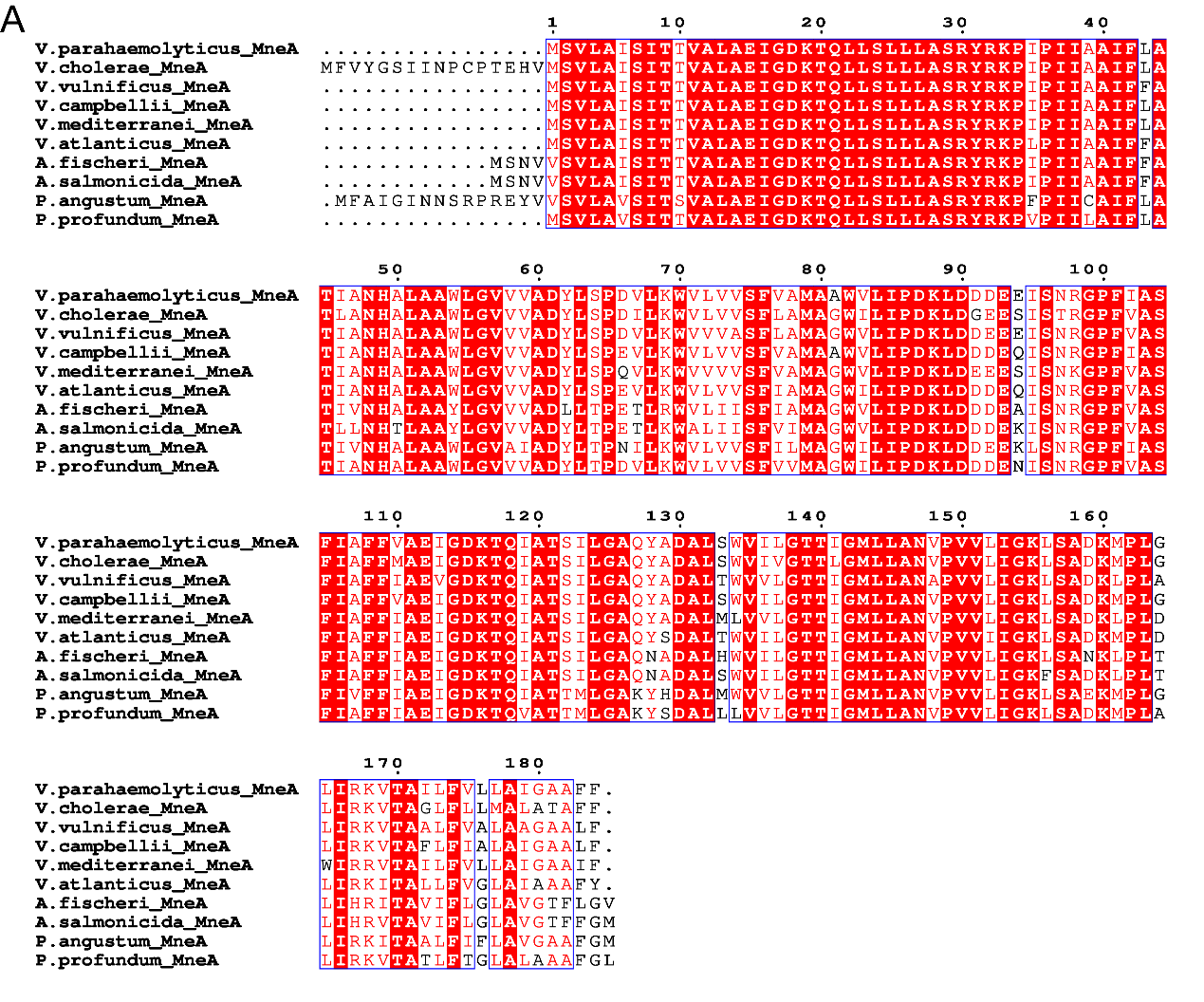


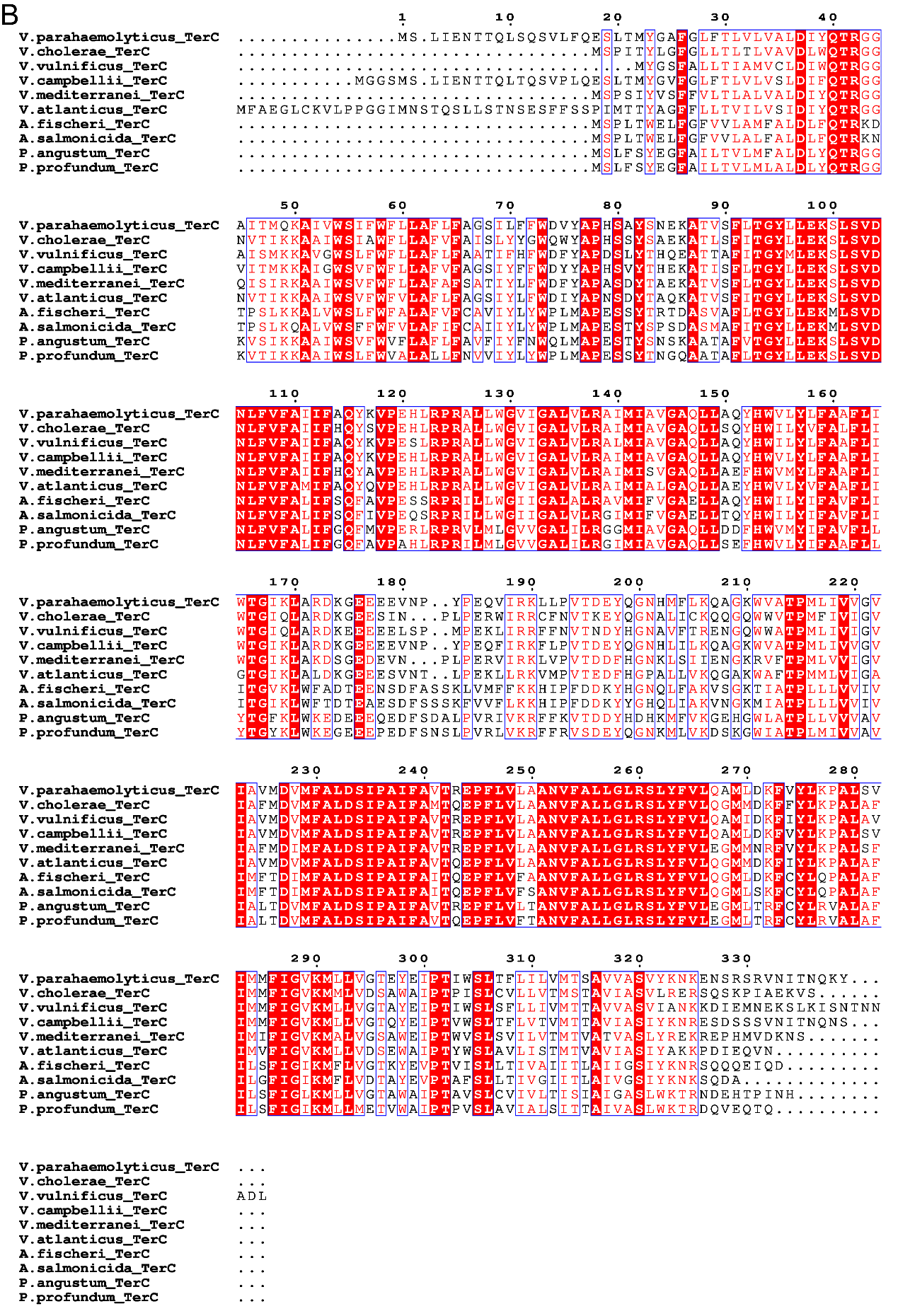


**Fig. S1.** Multiple sequence alignment of MneA (A) and TerC homologs (B) from *Vibrionaceae*. Multiple sequence alignment was performed using Clustal Omega, and the result was visualized using ESPript 3.0. The GenBank accession numbers are as follows: *V. parahaemolyticus* MneA, WP_005458690.1; *V. cholerae* MneA, WP_001884359.1; *V. vulnificus* MneA, WP_011079025.1; *V. campbellii* MneA, WP_010444803.1; *V. mediterranei* MneA, WP_006075101.1; *V. atlanticus* MneA, WP_009848160.1; *A. fischeri* MneA, WP_011263782.1; *A. salmonicida* MneA, WP_012552294.1; *P. angustum* MneA, EAS62829.1; *P. profundum* MneA, WP_011216894.1; *V. parahaemolyticus* TerC, WP_011106368.1; *V. cholerae* TerC, WP_000074622.1; *V. vulnificus* TerC, WP_011081212.1; *V. campbellii* TerC, WP_012129465.1; *V. mediterranei* TerC, WP_006073600.1; *V. atlanticus* TerC, WP_012600665.1; *A. fischeri* TerC, WP_011263168.1; *A. salmonicida* TerC, WP_012551468.1; *P. angustum* TerC, ZP_01235561.1; *P. profundum* TerC, WP_011218704.1.


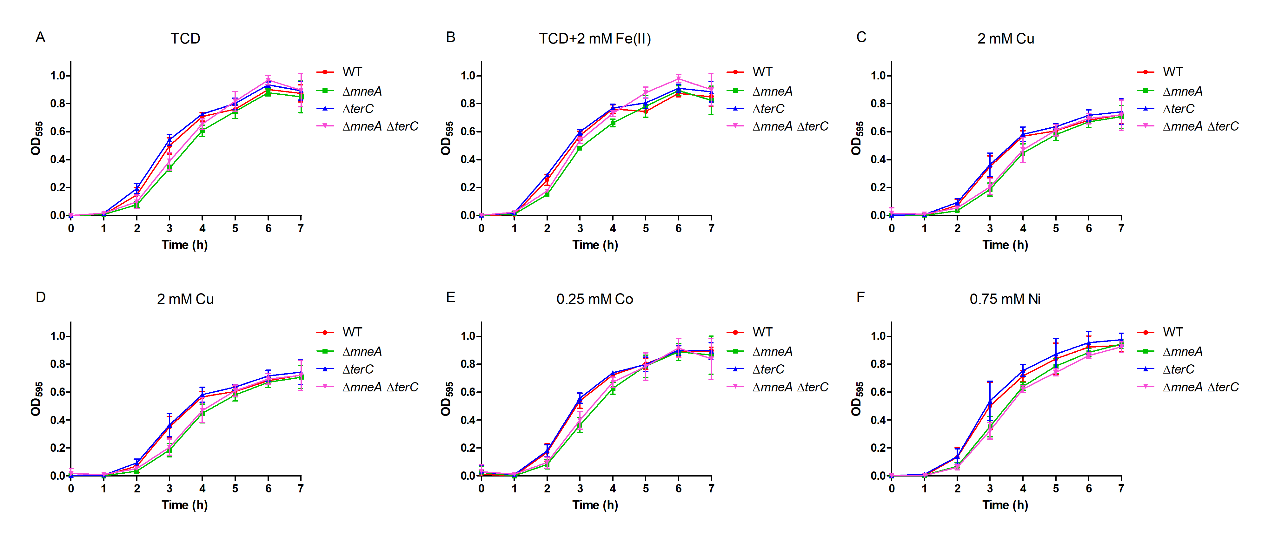


**Fig. S2.** The effect of added metals on the growth of the *V. parahaemolyticus* strains in standard TSB. The WT and mutant strains were grown in TSB supplemented with trisodium citrate dihydrate (TCD) (A), TCD plus 2 mM Fe(II) (B), 2 mM Zn (C), 2 mM Cu (D), 0.25 mM Co (E), or 0.75 mM Ni (F). The results represent the means and standard deviations from three independent experiments performed in duplicate.

**
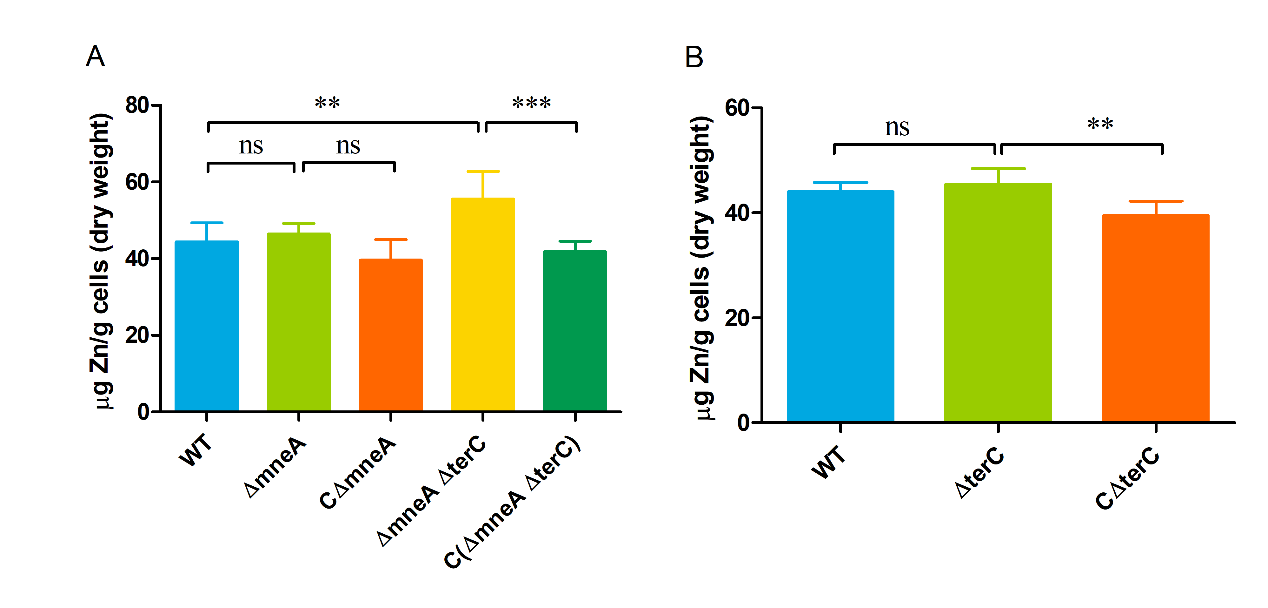
**

**Fig. S3.** ICP-MS analysis of intracellular Zn content in the *V. parahaemolyticus* strains cultured under Mn-supplemented conditions. (A) The intracellular Zn content in the WT, Δ*mneA*, CΔ*mneA*, Δ*mneA* Δ*terC*, and C(Δ*mneA* Δ*terC*) strains cultured in standard TSB supplemented with 25 μM Mn. (B) The intracellular Zn content in the WT, Δ*terC*, and CΔ*terC* strains cultured in TSB (pH 6.8) supplemented with 0.5 mM Mn. For the complemented strains, chloramphenicol (25 μg/mL) and IPTG (1 mM) were added to the medium to maintain the plasmid and induce gene expression. The WT and mutant strains were grown without chloramphenicol and IPTG. The results represent the means and standard deviations from six independent prepared samples. The data were analyzed using one-way analysis of variance along with Bonferroni’s post-test. **, *P*  <  0.01; ***, *P*  <  0.001; ns, no significant difference.


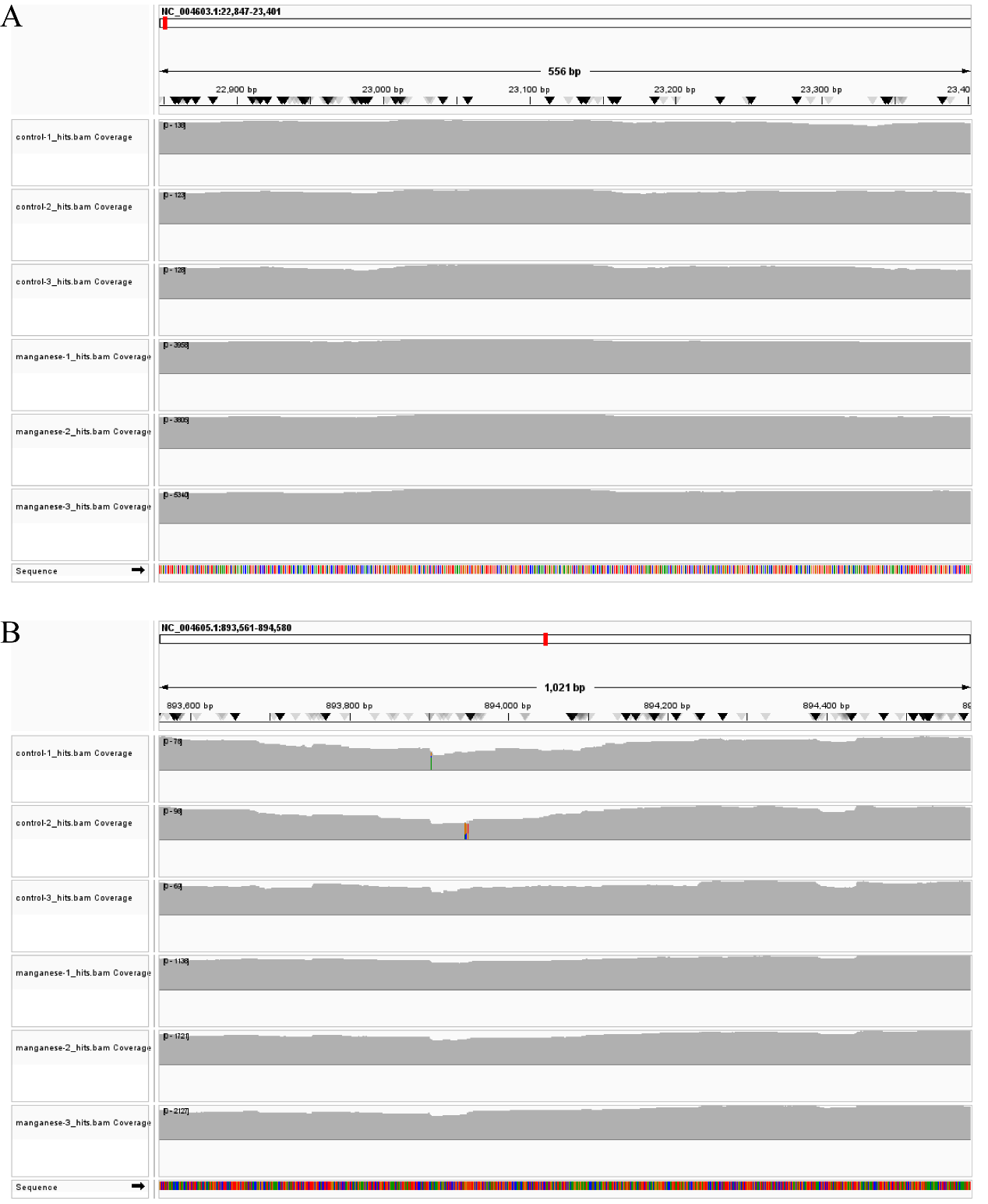


**Fig. S4.** RNA sequencing coverage of *mneA* (A) and *terC* (B) across coding regions under low-Mn (control) and Mn-supplemented conditions. *mneA* is located on chromosome 1 (NC_004603.1), positions 22847–23401, and *terC* is located on chromosome 2 (NC_004605.1), positions 893561–894580. The reads were mapped across the full coding sequences of both genes.


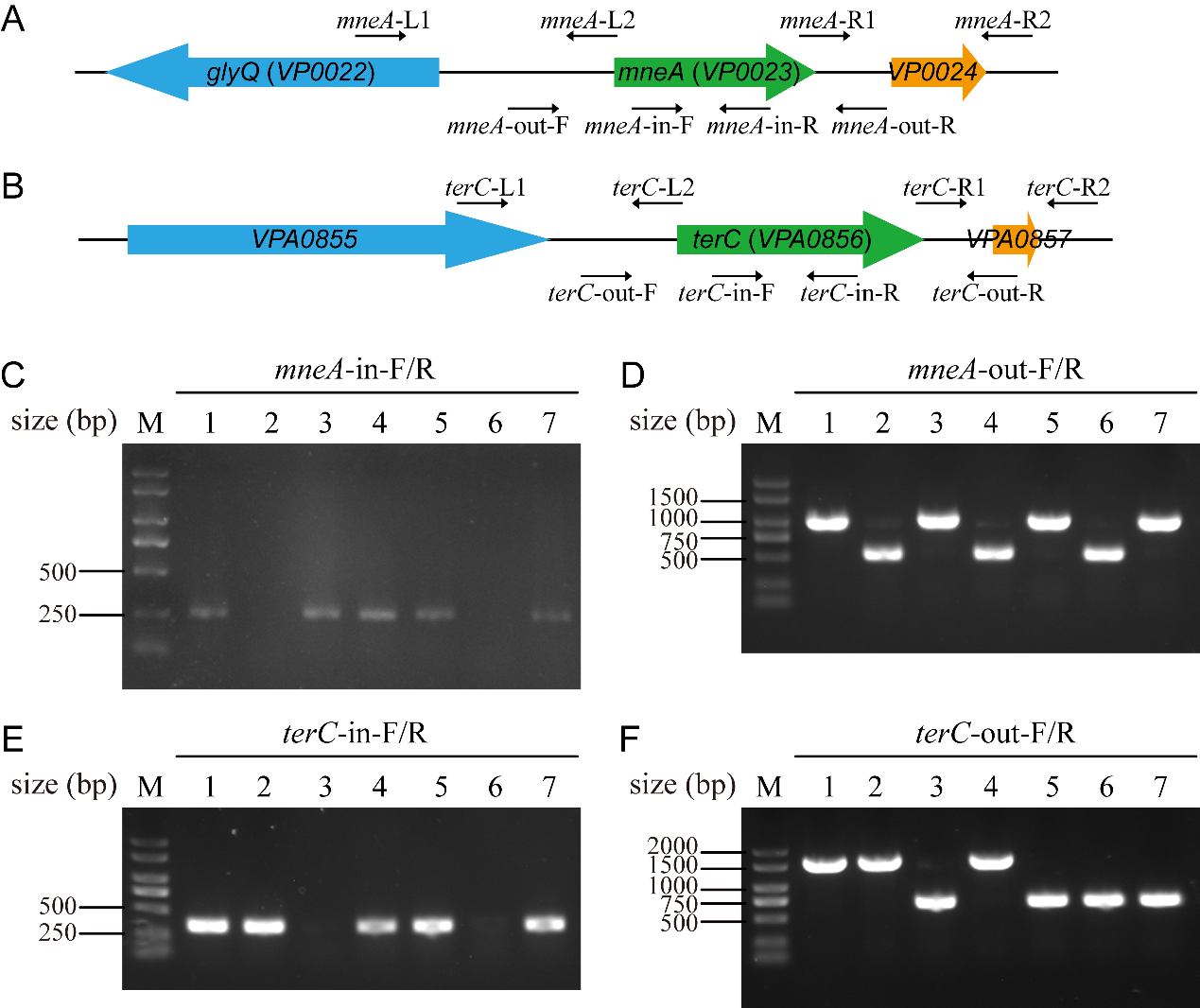


**Fig. S5.** PCR identification of mutant and complemented strains. (A-B) Positions of the primers used for the construction and identification of the Δ*mneA* (A) and Δ*terC* (B) mutants. (C-F) PCR amplification of genomic DNA from the WT strain (lane 1), Δ*mneA* (lane 2), Δ*terC* (lane 3), CΔ*mneA* (lane 4), CΔ*terC* (lane 5), Δ*mneA* Δ*terC* (lane 6), and C(Δ*mneA* Δ*terC*) (lane 7) using the primer pairs *mneA*-in-F/R (C), *mneA*-out-F/R (D), *terC*-in-F/R (E), and *terC*-out-F/R (F), respectively. Lane M: DL2000 DNA Marker.

**Table S2.** Bacterial strains and plasmids used in this study.

| Strain or plasmid | Relevant characteristics^a^ | Source or reference |
| --- | --- | --- |
| Strains |  |  |
| *V. parahaemolyticus* |  |  |
| RIMD2210633 | Clinical isolate; Carb^R^ | (1) |
| Δ*mneA* | *mneA* deletion mutant of RIMD2210633; Carb^R^ | This study |
| CΔ*mneA* | Complementation strain of Δ*mneA* (Δ*mneA* carrying pMMB207-*mneA*); Carb^R^, Cm^R^ | This study |
| Δ*terC* | *terC* deletion mutant of RIMD2210633; Carb^R^ | This study |
| CΔ*terC* | Complementation strain of Δ*terC* (Δ*terC* carrying pMMB207-*terC*); Carb^R^, Cm^R^ | This study |
| Δ*mneA* Δ*terC* | *mneA* and *terC* double mutant of RIMD2210633; Carb^R^ | This study |
| Δ*mneA* Δ*terC::mneA* | *mneA* complementation in Δ*mneA* Δ*terC*; Carb^R^ | This study |
| C(Δ*mneA* Δ*terC*) | Complementation strain of Δ*mneA* Δ*terC* (Δ*mneA* Δ*terC::mneA* carrying pMMB207-*terC*); Carb^R^, Cm^R^ | This study |
| *E. coli* |  |  |
| DH5α *λpir* | Cloning host for recombinant vector | Laboratory collection |
| S17-1 *λpir* | Conjugal donor for recombinant vector | Laboratory collection |
| Plasmids |  |  |
| pDM4 | Suicide vector containing a *sacB* counterselectable marker; Cm^R^ | (2) |
| pDM4-Δ*mneA* | Knockout vector for *mneA* deletion; Cm^R^ | This study |
| pDM4-Δ*terC* | Knockout vector for *terC* deletion; Cm^R^ | This study |
| pDM4-C*mneA* | pDM4 containing *mneA* and its flanking regions; Cm^R^ | This study |
| pMMB207 | Wide-host-range low-copy-number vector; Cm^R^ | (3) |
| pMMB207-*mneA* | pMMB207 containing *mneA* and an additional ribosome-binding site; Cm^R^ | This study |
| pMMB207-*terC* | pMMB207 containing *terC* and an additional ribosome-binding site; Cm^R^ | This study |

^a^ Carb^R^, carbenicillin resistant; Cm^R^, chloramphenicol resistant.

**Table S3.** Primers used in this study.

| Primer | Sequence (5'-3')^a^ | Size (bp) | Target gene | Application |
| --- | --- | --- | --- | --- |
| Q*mneA*-F | TTACTTGCCAGTCGTTACCGC | 108 | An internal region within the coding sequence of *mneA* (nucleotides 76–183) | qRT-PCR |
| Q*mneA*-R | GTCGGCCACTACCACACCTAAC |  |  |  |
| Q*terC*-F | ATTATGATTGCTGTCGGTGCG | 139 | An internal region within the coding sequence of *terC* (nucleotides 418–556) | qRT-PCR |
| Q*terC*-R | GCTCTGGGTATGGGTTCACTTCT |  |  |  |
| Q*gyrB*-F | GGTGGTATTCAAGCGTTCGTTC | 116 | An internal region of *gyrB* | qRT-PCR |
| Q*gyrB*-R | TGCATTGCCACTTCTACCGAG |  |  |  |
| *mneA*-L1 | TCC**CCCGGG**TCACCGTAACGGCCATC | 755 | The left arm of *mneA* | *mneA* deletion |
| *mneA*-L2 | CCAACAACACGTCTTATCGCCAATTTCTGC |  |  |  |
| *mneA*-R1 | gcgataagacGTGTTGTTGGCGATTGGT | 739 | The right arm of *mneA* |  |
| *mneA*-R2 | GC**TCTAGA**AGAGGCGGAAGGACTGC |  |  |  |
| *mneA*-in-F | TTCCAATTATTGCAGCCATC | 266 | An internal region of *mneA* | Identification of *mneA* deletion |
| *mneA*-in-R | AATGGAAGTCGCAATCTGAGT |  |  |  |
| *mneA*-out-F | GCTTTGACTCTTGTGATGCG | 1005/542 | A fragment containing *mneA* |  |
| *mneA*-out-R | ACTTGCCCTCTCGCTATCAT |  |  |  |
| C*mneA*-F | CGG**GGTACC**TAAGGAGGTAGGATAATAATGAGCGTTTTAGCTATTTCAAT | 555 | *mneA* and an additional ribosome-binding site | *mneA* complementation |
| C*mneA*-R | GC**TCTAGA**TTAAAAGAAGGCCGCACC |  |  |  |
| *terC*-L1 | TCC**CCCGGG**AGGCGAGGGTTGTGTGAG | 728 | The left arm of *terC* | *terC* deletion |
| *terC*-L2 | GCAACTACCGAGTGTGAAAAGGCCGAATG |  |  |  |
| *terC*-R1 | cttttcacactCGGTAGTTGCGTCGGTG | 752 | The right arm of *terC* |  |
| *terC*-R2 | GC**TCTAGA**GGTGGTCAGTCAATGTACGTG |  |  |  |
| *terC*-in-F | GTGCCAGAGCATCTTCGTC | 353 | An internal region of *terC* | Identification of *terC* deletion |
| *terC*-in-R | ATTGAATCGAGTGCAAACATC |  |  |  |
| *terC*-out-F | TGGGTAATGTGACTGCTGGT | 1533/679 | A fragment containing *terC* |  |
| *terC*-out-R | GCTGACAAATCTTCTGCGTAG |  |  |  |
| C*terC*-F | CGG**GGTACC**TAAGGAGGTAGGATAATAATGTCTCTTATCGAAAATACCAC | 1116 | *terC* and an additional ribosome-binding site | *terC* complementation |
| C*terC*-R | GC**TCTAGA**CTTGAATAACTTGGAGTTACAGG |  |  |  |

^a^ The bold sequences are restriction sites.

**References**

1. Makino K, Oshima K, Kurokawa K, Yokoyama K, Uda T, Tagomori K, Iijima Y, Najima M, Nakano M, Yamashita A, Kubota Y, Kimura S, Yasunaga T, Honda T, Shinagawa H, Hattori M, Iida T. 2003. Genome sequence of *Vibrio parahaemolyticus*: a pathogenic mechanism distinct from that of *V cholerae*. Lancet 361(9359):743-749.
2. Milton DL, O'Toole R, Horstedt P, Wolf-Watz H. 1996. Flagellin A is essential for the virulence of *Vibrio anguillarum*. J Bacteriol 178:1310-9.
3. Morales VM, Bäckman A, Bagdasarian M. 1991. A series of wide-host-range low-copy-number vectors that allow direct screening for recombinants. Gene 97:39-47.
